# Left extended hepatectomy with biliary resection and reconstruction for hilar cholangiocarcinoma in patient with Osler-Rendu-Weber disease: a case report and review of literature

**DOI:** 10.1007/s13304-025-02461-1

**Published:** 2025-11-25

**Authors:** Andrea Marchese, Simone Conci, Sara Pecori, Claudia Castelli, Alberto Contro, Giancarlo Mansueto, Aldo Scarpa, Andrea Ruzzenente

**Affiliations:** 1 Division of General and Hepatobiliary Surgery, Department of Surgical Sciences, Dentistry, Ginecology and Pediatrics, University of Verona Medical School, University Hospital G.B. Rossi, Piazzale L.A. Scuro, 10, 37134 Verona, Italy; 2 Division of Pathologic Anatomy and Histology, Department of Pathology and Diagnostics, University of Verona, University Hospital G.B. Rossi, 37134 Verona, Italy; 3 Department of Radiology, University of Verona, University Hospital G.B. Rossi, 37134 Verona, Italy

**Keywords:** Osler-Rendu-Weber syndrome, Extended hepatectomy, Bile duct resection, Peri-hilar cholangiocarcinoma, Interventional radiology

## Abstract

Osler-Rendu-Weber syndrome is a genetic disease that involves organs, liver included, characterized by alterations in the vessel walls, making them more vulnerable to spontaneous rupture and bleeding indeed. Our aim is to report a case of patient with Osler-Rendu-Weber syndrome undergoing extended hepatectomy with biliary resection for hilar cholangiocarcinoma and a review of literature on liver resection performed in patients with this syndrome. Preoperative, intraoperative, postoperative, radiographic, and pathologic data of case report’s patient were collected. Review of literature included studies from 2000 to 2024, searching them with following search keywords: (liver resection OR hepatectomy) AND (Osler-Rendu-Weber disease OR hereditary hemorrhagic telangiectasia). A 78-year-old woman with Osler-Rendu-Weber syndrome presented hilar lesion compatible with cholangiocarcinoma. Before surgery, the patient underwent embolization of an aneurysm in segment 6. A left extended hepatectomy with biliary resection was performed. Intraoperative blood loss was 500 cc. Post-operative course was uneventful and length of hospital stay was 10 days. 5 cases of liver resection in patient with this syndrome are reported in literature, including 2 cases of major hepatectomies. Major complications’ rate was 60% (3 cases): two cases of post-operative bleeding and one case of ascites decompensation. In one case exitus, consequent to massive bleeding, was reported (20%). This is the first case of extended hepatectomy with biliary resection performed in patient with Osler-Rendu-Weber syndrome. This underlying condition makes surgical approach demanding and challenging also in high volume centers. Proper patient selection and management could allow treatment and execution of a safe liver resection in patients with this syndrome.

## Introduction

Osler-Rendu-Weber syndrome (ORW), also called Hereditary hemorrhagic telangiectasia (HHT) is an autosomal dominant genetic disease characterized by the presence of vascular malformations (VMs) such as dilation of vascular structures, the presence of arterio-venous fistulas or pseudoaneurysms or telangiectasias affecting the skin, mucous membranes, lung, brain, gastro-intestinal tract and liver [1–6]. These VMs originated by alteration in the elastic and muscle layers of the vessel walls, making them more vulnerable to spontaneous rupture and injury. Therefore, the main symptom of ORW is hemorrhage from spontaneous vessels rupture.

In about 70% of patients with ORW, these VMs are present in the liver [5] and may create an imbalance between hepatic artery and portal venous blood flow that leads to an abnormal hepatic perfusion. The VMs and abnormal hepatic perfusion may cause endothelial damage with sinusoidal thrombosis and chronic regional hypoxia, leading to promotion of fibrosis. This hepatic environment may promote development of hepatocellular over-regenerative activity [7]. These particular and abnormal composition and structure of liver parenchyma has been supposed to be a risk factor for the development of liver regeneration nodules or liver lesions such as focal nodular hyperplasia (FNH) or hepatocellular carcinoma (HCC). Therefore, in a small subset of patients, there may be a need for hepatic resection [7–9].

In patients with ORW, the presence of vascular VMs and the state of hyperflux makes become liver resection technically complex and dangerous for increased risk of intra- and postoperative bleeding. Limited reports of liver cancers in patients with ORW are present in literature but there are very few reports on the results and outcomes of liver resection, mainly consisting in minor resections.

The aim of this manuscript is to report the case of a patient undergoing extended hepatectomy with biliary resection and reconstruction for peri-hilar cholangiocarcinoma in a patient with ORW and to report a review of the literature on liver resection performed in patients with ORW.

## Methods

### Case report

Preoperative, operative, postoperative, radiographic, and pathologic data of case report’s patient were collected.

### Review of literature

The review of literature was performed searching on PubMed, including studies from 2000 to 2024, and using the following search keywords: (liver resection OR hepatectomy) AND (Osler-Rendu-Weber disease OR hereditary hemorrhagic telangiectasia). ‘Related article’ function in PubMed was used to identify studies that may have been missed in the main research. Original clinical studies in English of any level of evidence were included.

## Results

### Case report

**Fig. 1:**
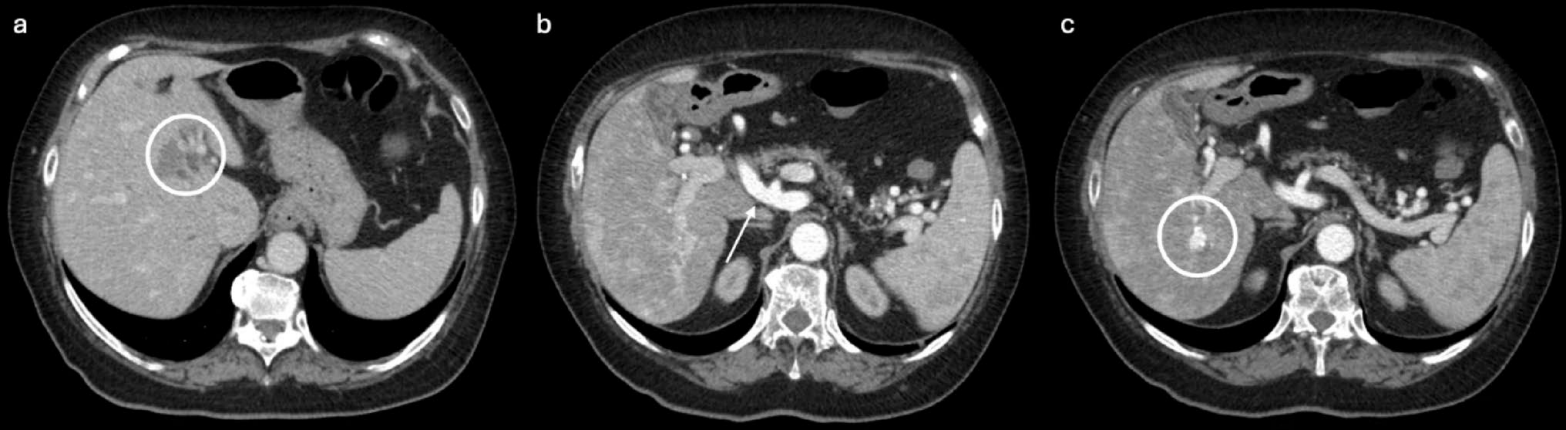
Patient’s CT scans. **A** Tumor in hilar region suspicious for hilar cholangiocarcinoma with dilation of intrahepatic bile ducts *(white circle)*. **B** Non-homogeneous parenchymography with multiple spots caused by intrahepatic telangiectasis. Intrahepatic aneurysm between segments 6 and 7 *(white circle)*. **C** Dilation of hepatic artery *(white arrow)*

A 78-year-old female patient with known Osler-Rendu-Weber disease (multiple episodes of epistaxis for nasal telangiectasia and cutaneous telangiectasia), presented abdominal pain, astheny and weight loss. Colonoscopy was performed and resulted in sigma diverticulosis. Blood exams showed elevated serum level of CA19.9 > 1000 KU/l. The CT scan identified a 25 mm hilar lesion invading the fourth segment, causing dilation of intrahepatic bile ducts and amputation of common bile duct. (Fig. 1A) The lesion was invading left hepatic artery and left portal vein. As collateral findings, the liver of the patient was characterized by multiple pseudo-nodular areas with enhancement in arterial phase compatible with telangiectasia, ectasia of celiac trunk and hepatic artery and intrahepatic aneurysm of 15 mm between segments 6 and 7. (Fig. 1B, C).

A CT-guided biopsy of the lesion was performed and resulted in a carcinoma with biliary immunophenotype.

Multidisciplinary discussion with radiologists and oncologists was performed. Due to the extension of disease requiring an extended hepatectomy to be performed with underlying ORW, the surgical risk of the patient was assessed as extremely high. Therefore, the patient was a candidate to chemotherapy with Cisplatine-Gemcitabine as a form of biologic selection in a high-risk surgical candidate. Unfortunately, only two cycles of therapy were given before suspension for pulmonary embolism. Therapy with Rivaroxaban was introduced.

After pulmonary embolism, surgical option was reconsidered. At blood exams, patient presented normal index of liver function and, surprisingly, a value of CA19.9 40 KU/l. A new multidisciplinary discussion was conducted with radiologists and oncologists and, considering the local extension of disease without extrahepatic disease, surgery was considered the best option.

**Fig. 2:**
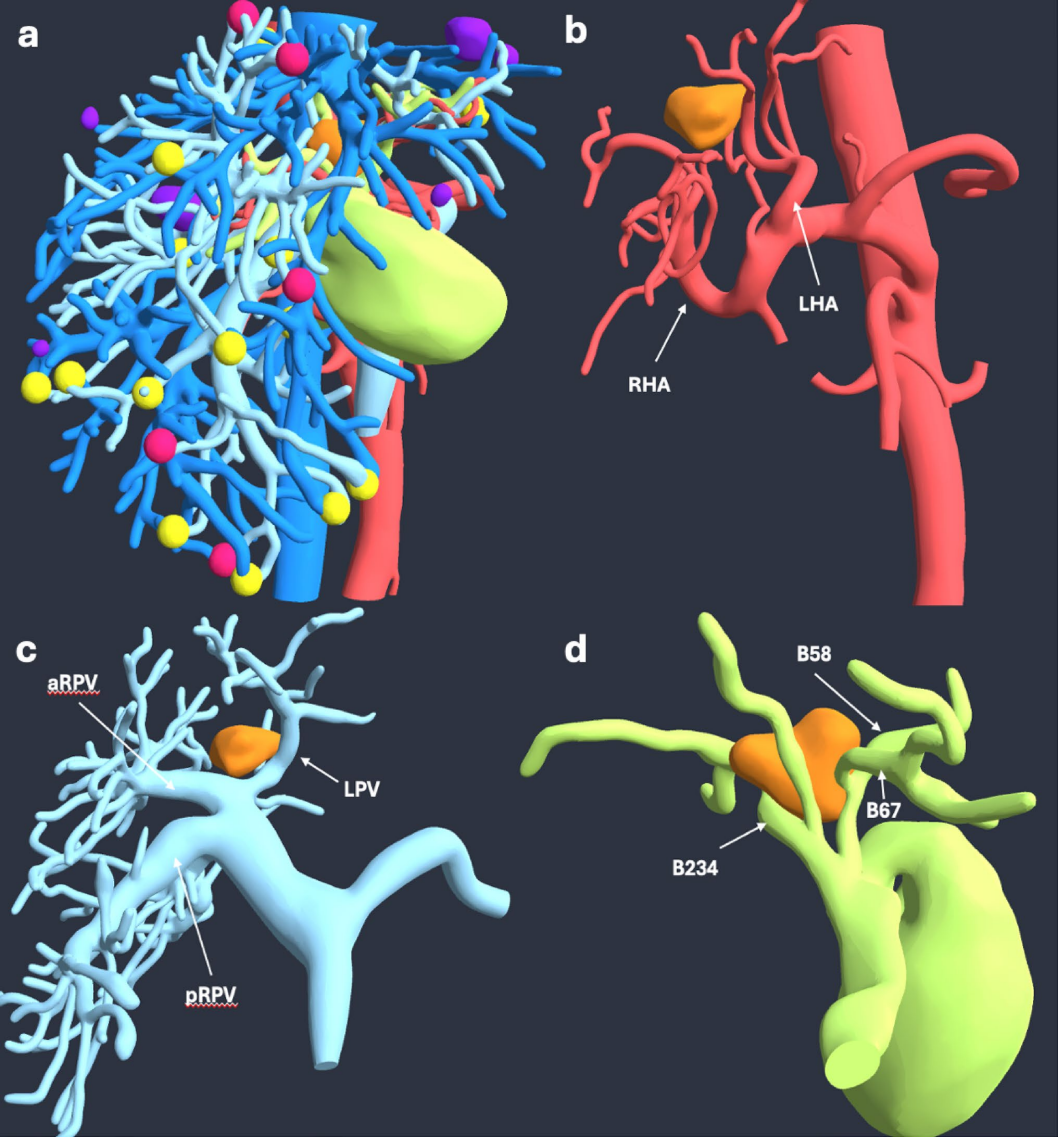
The three-dimensional visualization technology (3DVT) reconstruction. **A** Vascular and biliary structures of liver with multiple telangiectasis (yellow and pink spheres). **B** Arterial structures in anterior view. Dilation of the arteries and contact between the tumor (yellow) and the arterial branch for the fourth segment. **C** Portal structures in anterior view. Contact between the tumor (yellow) and the left portal vein (LPV) and the anterior right portal vein (aRPV). **D** Biliary tree in posterior view. Tumor (yellow) is in contact with left hepatic duct (B234) and the origin of right hepatic duct. It is shown a free margin on posterior right hepatic duct (B67)

The CT of the patient was reconstructed with 3D technology (HA3D™, MEDICS, Moncalieri, Turin, Italy) [10] that showed and confirmed the preoperative findings. The hilar lesion of 25 mm invaded the arterial branch for the fourth segment, the left portal vein and the separation of right anterior bile duct and right posterior bile duct (Fig. 2).

**Fig. 3:**
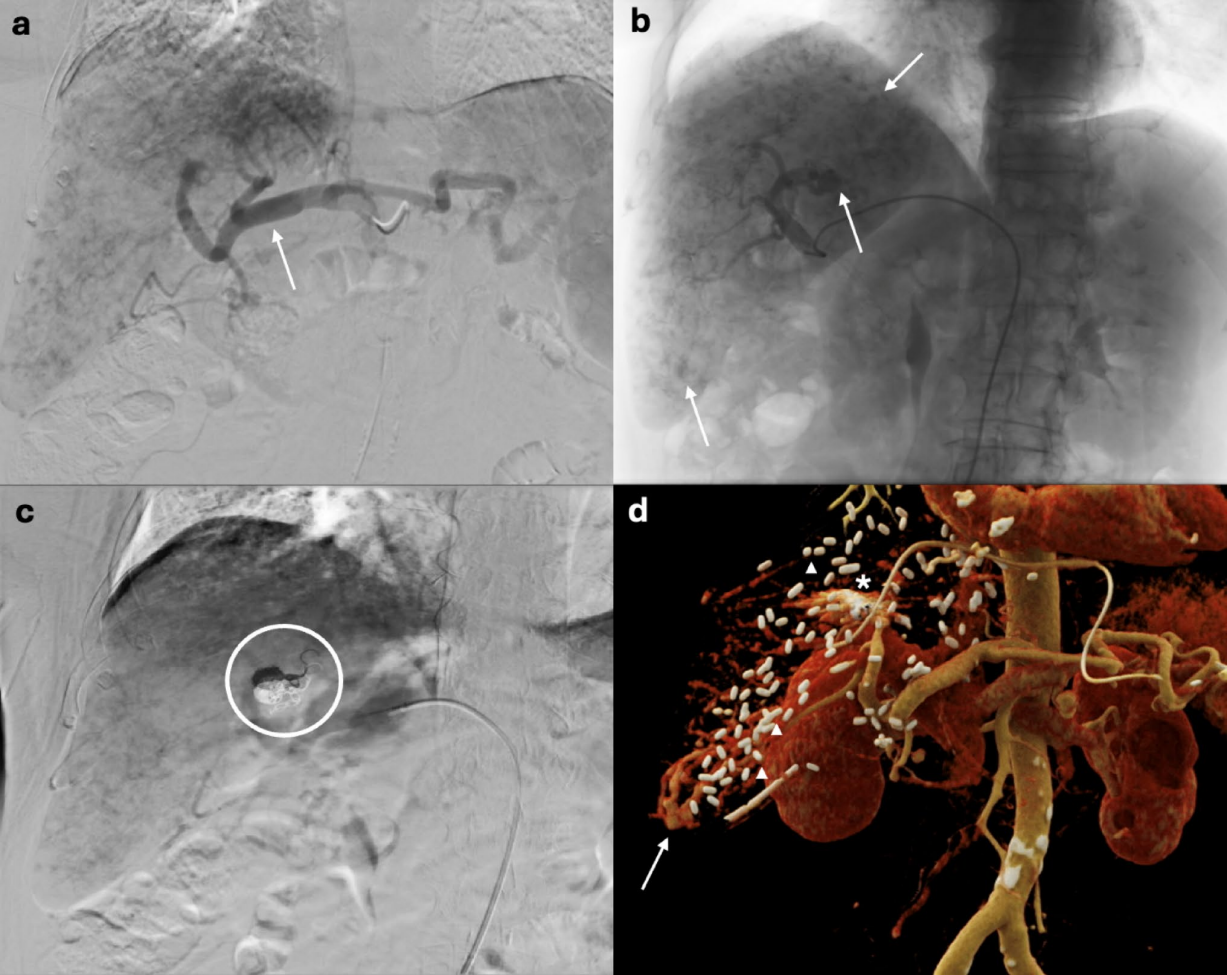
Patient’s angiography scans. **A** Hyperinflow in upper abdomen vascularization, causing multiple hepatic telangiectasia, ectasia of celiac trunk and hepatic artery *(white arrow)*. **B** Non-homogeneous parenchymography for the presence of multiple telangiectasis and intrahepatic aneurysm *(white arrows)*. **C** Embolization with spirals of intrahepatic aneurysm for segment 6 *(white circle).* **D** 3D rendering of remnant liver at post-operative day 7. Multiple medal clips are highlighted on transection line *(white arrowheads).* A preserved arterial vascularization is showed in the entire remnat liver despite embolization of aneurism for segment 6 *(asterisk)*. A persisting artero-venous fistula is highlighted *(white arrows)*

Considering the radial and longitudinal extension of disease, a left extended hepatectomy, caudate lobe resection and bile duct resection was indicated to reach radical resection. Future remnant liver volume (FRLV) for such extensive operation was 41% of the Total liver volume (TLV) and considered adequate. However, the presence of the intrahepatic aneurism in the remnant liver worried us. So, before conducting patient to the operating theater, the case was discussed with interventional radiologists to prevent future bleeding caused by intrahepatic aneurism between segments 6 and 7 (FRLV). A percutaneous angiography was performed and showed hyperinflux in upper abdomen vascularization, causing multiple hepatic telangiectasia, ectasia of celiac trunk and hepatic artery. The intrahepatic aneurism was embolized with metal spirals (AC, GM). The post-procedural course was uneventful. (Fig. 3A–C).

Afterwords, a left extended hepatectomy, resection of caudate lobe and bile duct resection was performed (H123458-B) [11]. Bilioenteric anastomosis was performed on single duct (B6-7). During lymphadenectomy and liver transection, hand-sewn ligatures were preferred to sealing device. Extemporaneous histological examination of proximal and distal bile ducts stumps did not show malignant infiltration. Intraoperative time and Pringle time were 570 min and 90 min, respectively. Intraoperative blood loss was 500 cc.

As internal protocol after major hepatectomies, the patient performed a CT scan at post-operative day 7 that, reported no post-operative abdominal collection or other complications and a regular vascularization of the remnant liver volume, despite the embolization of aneurism for segments 6 and 7. This could be explained by the optimal liver compensation due to the development, over the time, of numerous internal collateral circles (Fig. 3D).

Postoperative course was uneventful, length of hospital stay was 10 days.

**Fig. 4:**
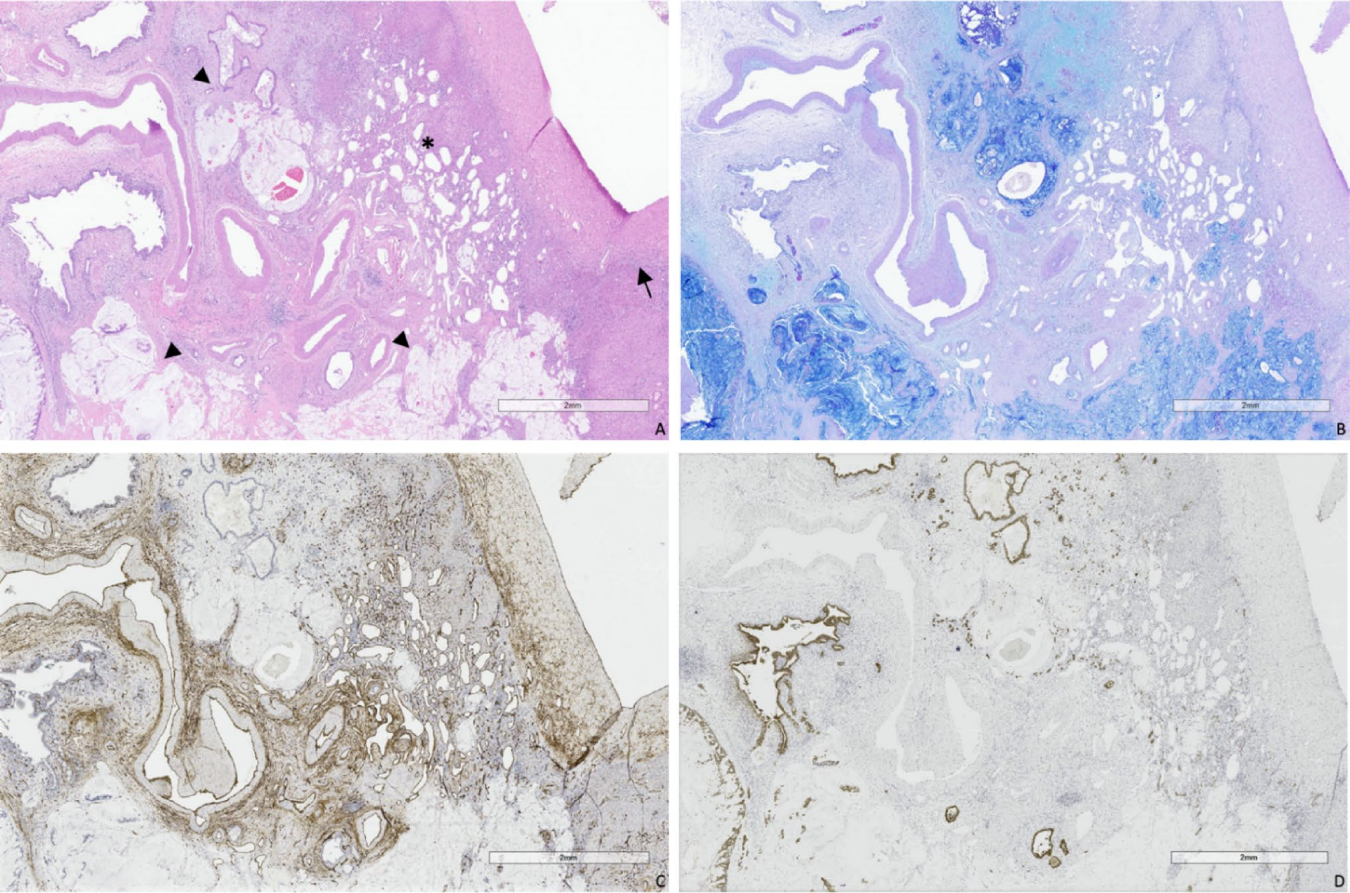
Representative image of cholangiocarcinoma (*arrowheads*) next to haphazard dilated sinusoidal channels (*asterisk*) and enlarged blood vessel (*arrow*), which are histologic features of hereditary hemorrhagic telangiectasia (Osler-Rendu-Weber syndrome). **A** Hematoxylin and eosin stain. **B** Alcian blue-PAS stain, that shows extracellular mucin component of the tumor. **C** Stain with CD34 antibody, which marks the endothelium of blood vessels. **D** Stain with cytokeratin 19 antibody, which is expressed by cancer cells and by normal biliary epithelial cells

The pathological exam of the surgical specimen demonstrated a suprahilar small-ducts cholangiocarcinoma, mucinous subtype, 25 mm in greatest dimension and infiltrating the biliary confluence. The tumor cells were positive for CK8-18, CK7, CK19 and partially for CDX2 and negative for CK20 and TTF-1. Resection margins were free of tumor cells (R0 resection). Six lymph-nodes were harvested and all of them were negative for metastasis. According to AJCC 8th edition, the neoplasm was a staged ypT4N0. The evaluation of the extralesional liver showed a preserved hepatic parenchymal architecture and the presence of blood vessels with enlarged caliber and sinusoidal dilatation, compatible with hereditary hemorrhagic telangiectasia. (Fig. 4).

After 12 months follow up the patient was disease free.

### Review of literature

During review of literature, 11 potentially relevant studies were found. 2 studies were rejected because regarding ORW patients’ generic management, 3 studies because reported cases of liver transplantation as main treatment of the disease, 1 study was rejected because reported a single case of treatment by interventional radiologists and 1 study was not considered in the review because reported only a vascular reconstruction without liver resection in patient with ORW. Thus, 4 studies were included in the review of literature, three case reports and one original article, reporting five liver resections in patients with ORW for both benign and malignant diseases. Morii et al. reported a case of anatomical resection of segment 6 for hepatocellular carcinoma with uneventful early post-operative course. At 8 months follow-up patient presented hepatic and lung recurrence disease and at 13 months exitus for massive hematemesis caused by a gastric angiodysplasia [7]. A case of anatomical resection of segment 6 for FNH was reported by Sekiguchi et al. [8]. The patient presented an uneventful early post-operative course but, as late complication, was reported a hematoma extended from liver section to pelvic pavement and treated with embolization of arterial branch to the segment 6 [8]. A patient with dyspnea and asthenia for arteriovenous malformation underwent a left lateral hepatectomy with uneventful post-operative course [12]. Gaujoux et al. reported two cases of liver malignancy in patient with ORW. The first patient underwent a left hepatectomy and lymphadenectomy for intrahepatic cholangiocarcinoma with post-operative course characterized by asymptomatic thrombosis of venous branch for seventh segment. In the second case was performed a right hepatectomy and wedge resection of third segment for colo-rectal liver metastasis with post-operative course characterized by ascites of nearly 1400 cc/die [13] (Table 1).

**Table 1:** Cases of liver resection in patients with ORW

Author (year)	Liver Lesion	Liver resection*	Intraoperative blood loss (cc)	Post-operative course	LOS (days)	Follow-up (months)
Orlando [12]	MAV	H23	–	Uneventful	5	48
Gaujoux [13]	iCCA	H234 + Lymphadenectomy	800	Venous thrombosis of branch for Sg7	–	–
	CRLM	H5678/H3’	800	Ascites (1400 cc/die)	–	–
Morii [7]	HCC	H6	–	Delayed massive hematemesis from gastro-intestinal angiodysplasia with exitus	–	13
Sekiguchi [8]	FNH	H6	220	Hematoma from liver section to pelvis treated with embolization of arterial branch for Sg6	14	12
	pCCA	H123458-B	500	Uneventful	10	12

*MAV* Arterovenous Malformations, *iCCA* intrahepatic cholangiocarcinoma, *CRLM* colo-rectal liver metastasis, *HCC* hepatocellular carcinoma *FNH* focal nodular hyperplasia *pCCA* peri-hilar cholangiocarcinoma, *LOS* length of hospital stay

*According to “New World Terminology”

## Discussion

In ORW the weak vascular wall predisposes to possible ruptures resulting in bleeding. For this reason, liver resection can be challenging even for the most experienced surgeons.

The state of increased arterial and portal flow seems to be related to changes in the parenchyma with increased risk of portal hypertension, fibrosis and then cirrhosis. Thus, liver resection with this underlying condition may predispose to an increased risk of post-hepatectomy liver failure (PHLF) or ascitic decompensation. Gaujoux et al. reported an ascitic decompensation of about 1400 cc/day after major hepatectomy solved with conservative treatment [13]. It seems therefore an additional characteristic to consider while performing a liver resection in patient with ORW. In our case, there was no ascitic decompensation of the patient. It is probably due to the sufficient remnant liver volume for a left extended hepatectomy (41%), the optimal initial liver function of the patient and a normal extra-lesional structure of the liver parenchyma at pathological findings.

VMs in ORW predispose to spontaneous bleeding as shown by the multiple episodes of epistaxis present in these patients [14]. In our case, multiple episodes of epistaxis were reported. This condition of weak vascular structures leads to an increased risk of postoperative bleeding in patients. In literature, two cases out of five reported post-operative bleeding (40%) and one of them led to the exitus of the patient (20%). Morii et al., although they showed a regular postoperative course, reported the patient’s exitus for massive hematemesis from gastro-intestinal angiodysplasia rupture [7]. Sekiguchi et al. reported an interventional management of post-operative bleeding from liver stump that formed a massive pelvic hematoma [8]. In our case the patient did not present postoperative bleeding.

In patient with ORW candidate to liver resection, the main purpose of surgeons may be to pursue a “safe liver resection” as much as possible. Regarding the initial management and selection of the patient, it should be mandatory to consider the initial liver function and the future remnant liver volume to avoid the high risk of PHLF characterizing this cohort of patients. Moreover, the intraoperative strategies to reduce the risk of postoperative bleeding should be pursued. In our experience, routinely in open approach, the minor vessels are sealed with radiofrequency sealing devices. Hand-sewn ligatures are performed to only close glissonian pedicles. Due to high-risk bleeding patient and vascular fragility, this approach seemed to be insufficient to pursue a “safe liver resection”. As reported by Morii et al. [7] it might be useful to refer to hand-sewn ligatures for vessels that are found during resection rather than using energy devices. This is because after the hepatic resection, the hyperflux condition present in the hepatic parenchyma predisposes to even late bleeding as soon as the eschar falls. Therefore, in our case, hand-sewn ligatures were used throughout the resection and during the resection of the main bile duct, not showing any intra- and post-operative bleeding.

Although, the alterations in the intrahepatic circulation are supposed to be predisposing the formation of primitive lesions of the liver as HCC or FNH, no clear correlation are reported between hepatic ORW and the onset of biliary tract cancers (BTCs). Only another case of BTCs has been previously reported by Gaujoux et al. [13], without evidence of a definitive relationship. Further research will be needed to investigate the correlation between diseases.

To our knowledge this is the first reported case of hepatic resection associated with resection of the main bile duct for hilar cholangiocarcinoma in patients with underlying ORW. Our experience of performing a complex liver resection with bile duct resection could demonstrate the feasibility of demanding surgical treatment in this high-risk cohort of patients but relegated to tertiary referral center.

## Conclusions

The ORW predisposes patients undergoing liver resection to an increased risk of post-operative complications such as liver decompensation or bleeding (40% of cases reported in literature). This condition makes the surgical approach to these patients demanding and challenging also in high volume centers and require a multidisciplinary discussion and approach. Proper patient selection and management could allow treatment and execution of a safe liver resection in patients with ORW.
